# Transcriptional profiling of two different physiological states of the yak mammary gland using RNA sequencing

**DOI:** 10.1371/journal.pone.0201628

**Published:** 2018-07-30

**Authors:** Fan Jiangfeng, Luo Yuzhu, Yu Sijiu, Cui Yan, Xu Gengquan, Wang Libin, Pan Yangyang, He Honghong

**Affiliations:** 1 College of Veterinary Medicine, Gansu Agricultural University, Lanzhou, Gansu, P. R. China; 2 College of Animal Science and Technology, Gansu Agricultural University, Lanzhou, Gansu, P. R. China; 3 Technology and Research Center of Gansu Province for Embryonic Engineering of Bovine and Sheep & Goat, Lanzhou, Gansu, P. R. China; University of Illinois, UNITED STATES

## Abstract

Yak milk is superior to common cow milk in nutrients including protein, fat and calories. However, the milk yield of the yak is very much lower compared with other dairy bovines. To understand the molecular mechanisms of lactogenesis, lactation and mammary gland development, mammary tissue samples were taken from five yaks during a dry period (DP, n = 3) and lactation period (LP, n = 2). Two types of cDNA sequence libraries that reflected the different physiological states of the mammary gland were constructed using RNA sequencing technology. After removing reads containing adapters, reads containing poly-N and low-quality reads from the raw data, 45,423,478 to 53,274,976 clean reads were obtained from these libraries. A total of 74.72% to 80.65% of the high-quality sequence reads were uniquely aligned to the BosGru v2.0 yak reference genome. Using the DESeq R package, 360 differentially expressed genes were detected between the two groups when the adjusted P value (padj < 0.05) was used as the cutoff value; this included 192 upregulated and 168 downregulated genes in the yak mammary gland tissue of the DP compared to the LP. A gene ontology analysis revealed that the most enriched GO terms were protein binding, multi-organism process, immune system and others. KEGG pathway analysis indicated that the differentially expressed genes were mostly enriched in Hippo signaling, insulin signaling, steroid biosynthesis and others. The analysis of the up- and downregulated genes provides important insights into the molecular events involved in lactogenesis, lactation and mammary gland development and will guide further research to enhance milk yield and optimize the constituents of yak milk.

## Introduction

The yak (*Bos grunniens*) is a specific type of livestock that is mainly distributed in the Qinghai-Tibetan Plateau and adjacent regions. It occupies a very important and irreplaceable ecological and economical position in local regions. It has also been regarded as an iconic symbol of Tibet and high altitudes. At present, there are approximately 14 million yaks in the world, of which more than 90% are in China [1]. The yak is an abundant source of milk, meat and other necessities for Tibetans. Yak milk is also the raw material of local residents’ traditional food, such as milk tea, butter, cheese and others. Yak milk is superior to common cow milk in nutrients including protein, fat and calories. However, the yak has a much lower milk yield compared with other dairy bovines due to conditions of extreme harshness, including extreme cold and low air pressure and oxygen content with high solar radiation at high altitudes in the Tibetan Plateau. The milk yield of a yak is usually 200~300 kg, the lactation duration is just 150~200 d, and the daily milk yield is commonly 1.5~2.0 kg [2–4]. The very low milk yield has confused researchers for a long time. A reasonable approach to improving the milk yield of the yak would be to investigate the key genes and understand the regulatory mechanism of lactation.

High-throughput mRNA sequencing (RNA-seq) offers the ability to survey the genes and transcripts that are expressed in a biological sample under a specific physiological or pathological condition. It also provides a far more precise measurement of the levels at which genes and transcripts are expressed compared to other methods [4,5]. A large number of investigations have revealed the transcriptional profiles of different organs and tissues of various livestock using RNA-seq technology in recent years [4]. In the yak, RNA-seq was used to construct a tissue-specific cDNA library and survey the important regulatory genes related to reproduction [6], coat color [7] and others. Regarding the physiological process of lactation, many studies have been conducted on the mammary gland or milk of bovine [2,8–14] and other animals [15–17]. In addition, Wang et al. [9] identified 517 annotated differentially expressed genes in mammary tissues from three yaks at 1 and 30 d after parturition using Affymetrix Bovine Genome Arrays. However, beyond that, limited research has been published on the detailed characterization of global genes expressed in the yak mammary gland. In the present study, we used RNA sequencing (RNA seq) technology to examine the genes expressed in the mammary gland of the yak at a dry period (DP) and a lactation period (LP). These data may provide candidate genes with a high probability of having functional roles in regulating the lactation process in the yak mammary gland and providing a theoretical basis for the improvement of the milk yield of the yak.

## Materials and methods

### Animals

All procedures involving animals were approved by the Animal Care and Use Committee of Gansu Agricultural University where the experiment was conducted. Five female yaks at 6~8 years-of-age were selected and raised in Xiahe County (Gansu Province, China) by a local herdsman. These yaks were raised under a similar environment with natural grazing and had free access to water with no supplementary feeding. The yaks were divided into two groups. Three of them (DP1, DP2, DP3) were in the dry period (DP group: calved and ablactated in the winter of the last year with no pregnancy and no milking at the time of sample collection) and two of them (LP1, LP2) were in the lactation period (LP group: calved in the spring of the current year and were suckling the calf at the time of sample collection). The selected female yaks were slaughtered in June 2016, and the mammary gland tissues were gathered and immersed into liquid nitrogen within 20 min after death.

### Total RNA isolation and quality evaluation

Total RNA was isolated from each tissue sample using Trizol reagents (Cat. No. 15596018, Invitrogen, Carlsbad, CA) according to the manufacturer’s instructions. The integrity of the isolated RNA was assessed using the RNA Nano 6000 Assay Kit of the Bioanalyzer 2100 system (Agilent Technologies, CA,USA), and the concentration of RNA was measured using the Qubit® RNA Assay Kit (Cat. No. Q32852, Invitrogen, USA) in a Qubit® 2.0 Fluorometer (Life Technologies, USA).

### Library preparation and RNA sequencing

In the present study, 3 μg of RNA per sample and the NEBNext® Ultra™ RNA Library Prep Kit for Illumina® (NEB, USA) were used to generate the sequence libraries according to the manufacturer’s recommendations. In brief, after purification from total RNA using poly-T oligo-attached magnetic beads, mRNA was split into fragments using divalent cations under an elevated temperature in NEBNext First Strand Synthesis Reaction Buffer(5X). Then, first strand cDNA was synthesized using M-MuLV Reverse Transcriptase(RNase H-)and random hexamer primers. Subsequently, second strand cDNA was synthesized using DNA Polymerase I and RNase H. The remaining overhangs were converted into blunt ends via exonuclease/polymerase activities. After adenylation of the 3’ ends of DNA fragments, NEBNext Adaptors with a hairpin loop structure were ligated for further hybridization. The library fragments were purified with the AMPure XP system (Beckman Coulter, Beverly, USA) to preferentially select cDNA fragments of 150~200 bp in length. Then, 3 μl of USER Enzyme (NEB, USA) was used with size-selected adaptor-ligated cDNA at 37°C for 15 min followed by 5 min at 95°C before PCR. Then, PCR was performed with Universal PCR primers, Index (X) Primer and Phusion High-Fidelity DNA polymerase. Finally, after PCR products were purified (AMPure XP system) and library quality was assessed on the Agilent Bioanalyzer 2100 system, the library preparations were sequenced on an Illumina Hiseq platform at Beijing Novogene Bioinformatics Technology Co., Ltd. (Beijing, China).

### Read mapping

Reference genome and gene model annotation files of the yak (BosGru v2.0, https://www.ncbi.nlm.nih.gov/assembly/GCF_000298355.1/) were downloaded from the genome website directly. An index of the reference genome was built using Bowtie v2.2.3, and paired-end clean reads were aligned to the reference genome using TopHat v2.0.12. TopHat was selected as the mapping tool because it can generate a database of splice junctions based on the gene model annotation file and thus provide a better mapping result than other non-splice mapping tools.

### Quantification of gene expression levels

HTSeq v0.6.1 was used to count the reads numbers mapped to each gene. Then, the expected number of Fragments Per Kilobase of transcript sequence per Millions base pairs sequenced (FPKM) of each gene was calculated based on the length of the gene and reads count mapped to this gene. Considers the effect of sequencing depth and gene length for the reads count at the same time, FPKM is currently the most commonly used method for estimating gene expression levels [18].

### Differential expression analysis

A differential expression analysis between the DP and LP was performed using the DESeq R package (1.18.0). DESeq provides statistical methods for determining differential expression in digital gene expression data using a model based on the negative binomial distribution. The resulting P values were adjusted using the Benjamini and Hochberg’s approach for controlling the false discovery rate. Genes with an adjusted P value <0.05 found by DESeq were assigned as differentially expressed.

### Annotations of differentially expressed genes

To extract biological meanings from differentially expressed genes, a gene enrichment analysis was performed with Gene Ontology (GO) [19] and Kyoto Encyclopedia of Genes and Genomes databases (KEGG, http://www.genome.jp/kegg) [20,21].

## Results

### Summary of the sequence reads

In the present study, two types of cDNA sequence libraries were constructed using mammary tissues of the dry period (DP) and lactation period (LP) from the yak. A total of 47,006,966 to 54,251,010 raw reads were generated for each sample. After removing reads containing adapters, reads containing poly-N and low-quality reads from raw data, 45,423,478 to 53,274,976 clean reads were obtained. At the same time, the Q20, Q30 and GC content of the clean data were calculated. The results indicated that the base composition of the raw sequence data was satisfactory and the ratio of the low-quality base sequence reads (<20) was very low (Table 1). All the downstream analyses were based on the clean reads with high quality.

**Table 1:** The data and qualities of RNA sequencing for five samples.

Sample name	Raw reads	Clean reads(%)	Low Quality(%)	Error rate(%)	Q20(%)	Q30(%)	GC content(%)
DP1	54251010	53274976(98.20)	210004(0.77)	0.02	97.02	92.52	50.46
DP2	49403752	47465200(96.08)	209844(0.85)	0.02	96.15	90.4	50.41
DP3	47006966	45423478(96.63)	227761(0.97)	0.02	95.99	90.08	50.26
LP1	52443546	50653414(96.59)	195546(0.75)	0.02	96.3	90.69	50.53
LP2	50133478	49336866(98.41)	203310(0.81)	0.02	97.17	92.8	47.95

Using TopHat v2.0.12, 76.85% to 84.56% of the total reads were aligned to the BosGru v2.0 (https://www.ncbi.nlm.nih.gov/assembly/GCF_000298355.1/) yak reference genome and 74.72% to 80.65% of the reads were uniquely mapped to the yak genome for each sample. The statistics results indicated that the percentage of reads mapped to the yak reference genome exon was over 52.5% in all these samples (Table 2).

**Table 2:** The summary of reads mapped to the yak reference genome (BosGru v2.0).

Sample_name	DP1	DP2	DP3	LP1	LP2
Total reads	53274976	47465200	45423478	50653414	49336866
Total mapped	42253621 (79.31%)	36591074 (77.09%)	34907805 (76.85%)	39887510 (78.75%)	41718425 (84.56%)
Multiple mapped	1042587 (1.96%)	882264 (1.86%)	967422 (2.13%)	1111091 (2.19%)	1929306 (3.91%)
Uniquely mapped	41211034 (77.36%)	35708810 (75.23%)	33940383 (74.72%)	38776419 (76.55%)	39789119 (80.65%)
Reads map to '+'	20606327 (38.68%)	17852854 (37.61%)	16973995 (37.37%)	19337127 (38.18%)	19754080 (40.04%)
Reads map to '-'	20604707 (38.68%)	17855956 (37.62%)	16966388 (37.35%)	19439292 (38.38%)	20035039 (40.61%)
Non-splice reads	26939899 (50.57%)	22706838 (47.84%)	21256646 (46.8%)	22197598 (43.82%)	18208947 (36.91%)
Splice reads	14271135 (26.79%)	13001972 (27.39%)	12683737 (27.92%)	16578821 (32.73%)	21580172 (43.74%)
Mapped to Exon(%)	52.5	54.1	56.2	63.8	75.1
Mapped to Intron(%)	13.6	13.9	9.8	7.6	5.1
Mapped to Intergenic(%)	33.8	32.0	34.0	28.6	19.8

An additional analysis using rMATS (http://rnaseq-mats.sourceforge.net/index.html) showed that there were two major alternative splice (AS) patterns in the yak transcriptome data, which included skipped exons (SEs) and mutually exclusive exons (MXEs). A total of 10,924 SE events were found in all these samples using Junction Counts plus read on target detection, in which nine SE events were different (six events were upregulated, and 3 events were downregulated in the yak mammary tissues of the DP compared with that of the LP). The statistics of the alternative splice (AS) types and amount is showed in Table 3.

**Table 3:** The statistics of alternative splice (AS) types and amount.

Event Type	NumEvents.JC.only	SigEvents.JC.only	NumEvents.JC+readsOnTarget	SigEvents.JC+readsOnTarget
SE	10904	9 (6:3)	10924	9 (6:3)
MXE	911	0 (0:0)	911	0 (0:0)
A5SS	0	0 (0:0)	0	0 (0:0)
A3SS	0	0 (0:0)	0	0 (0:0)
RI	0	0 (0:0)	0	0 (0:0)

### Quantification of gene expression level

**Fig 1:**
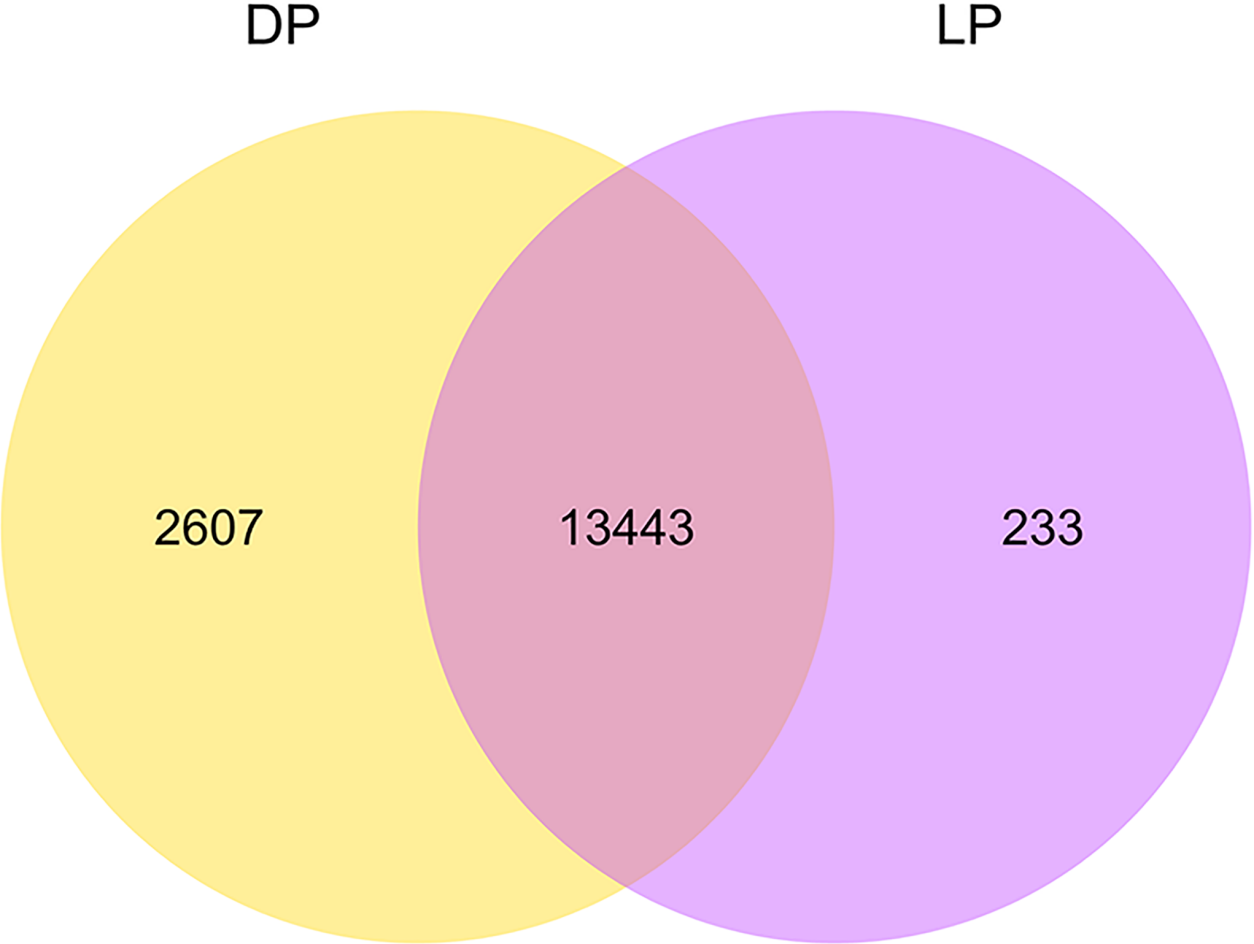
A venn diagram of the amounts of the expressed genes in two groups.

**Fig 2:**
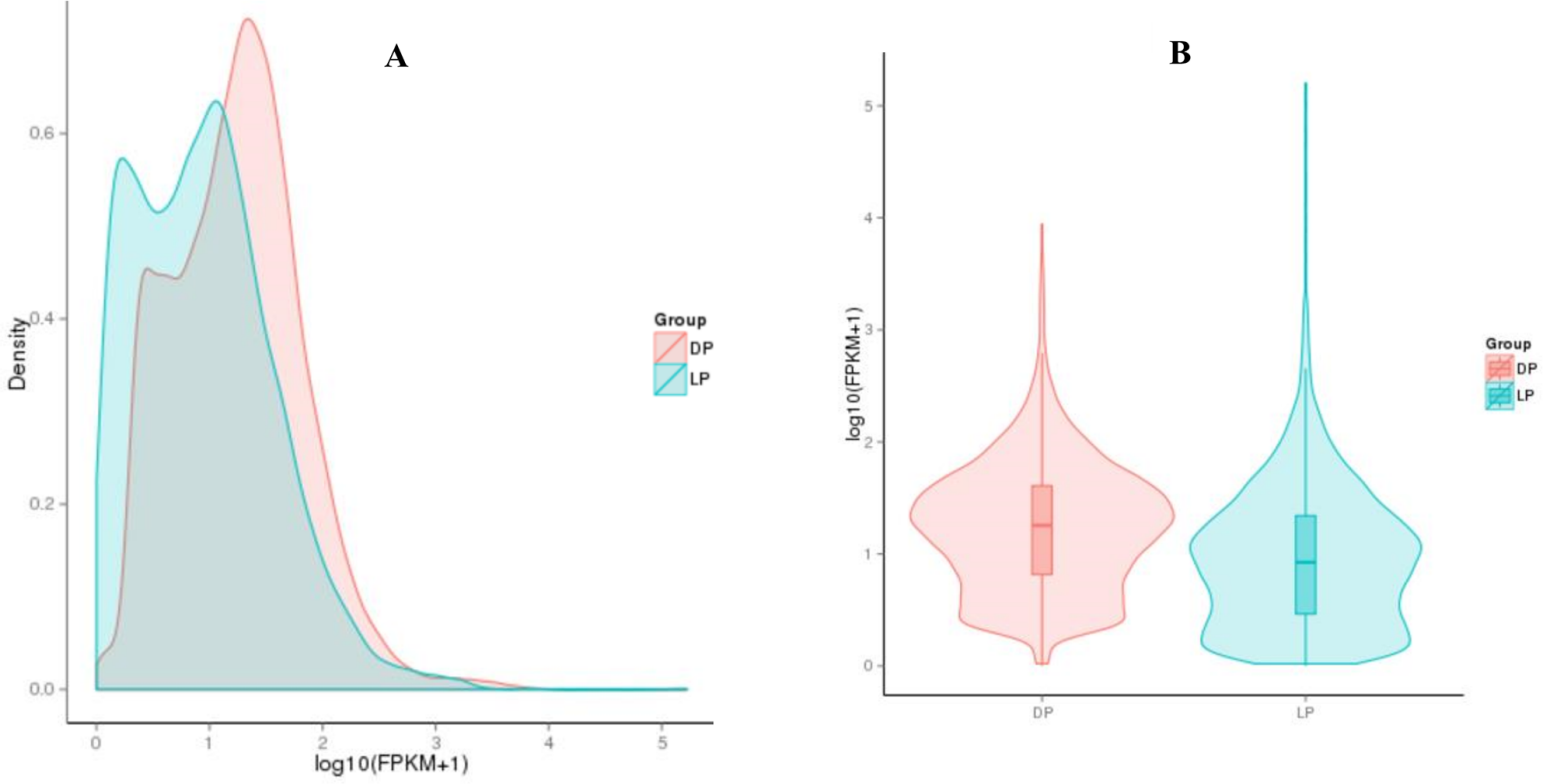
The Density Distribution (A) and Violin (B) Diagram of the FPKM in Two Groups.

In the present study, HTSeq v0.6.1 was used to quantify the expression level of mapped genes in each sample, and the FPKM (expected number of Fragments Per Kilobase of transcript sequence per million base pairs sequenced) value of each gene was calculated based on the length of the gene and reads count mapped to this gene. In our analysis, the number of expressed genes (FPKM values > 1.0) ranged from 12,402 (49.85%) to 16,068 (64.59%) in all five samples. Among these genes, 793 (3.19%) to 2632 (10.58%) genes were highly expressed with FPKM values > 60.0. The statistics of gene distribution at different expression levels is shown in Table 4. Additional analyses indicated that 13,443 genes were expressed in both the DP and LP; furthermore, 2,607 and 233 genes were specifically expressed in the DP and LP, respectively (mean FPKM value > 1.0). We also show a Venn diagram (Fig 1), density distribution (Fig 2A) and violin diagram (Fig 2B) of FPKM to visually demonstrate the expression level of all the genes in the yak mammary gland tissues of the dry period (DP) compared to those of the lactation period (LP).

**Table 4:** The statistics of genes at different expression levels.

FPKM Interval	DP1	DP2	DP3	LP1	LP2
0~1	8809(35.41%)	9073(36.47%)	9090(36.54%)	10715(43.07%)	12475(50.15%)
1~3	2280(9.17%)	2229(8.96%)	2245(9.02%)	2350(9.45%)	3022(12.15%)
3~15	5129(20.62%)	4960(19.94%)	5047(20.29%)	5275(21.20%)	6043(24.29%)
15~60	6133(24.65%)	6035(24.26%)	5863(23.57%)	4381(17.61%)	2544(10.23%)
>60	2526(10.15%)	2580(10.37%)	2632(10.58%)	2156(8.67%)	793(3.19%)
>1	16068(64.59%)	15804(63.53%)	15787(63.46%)	14162(56.93%)	12402(49.85%)

**Fig 3:**
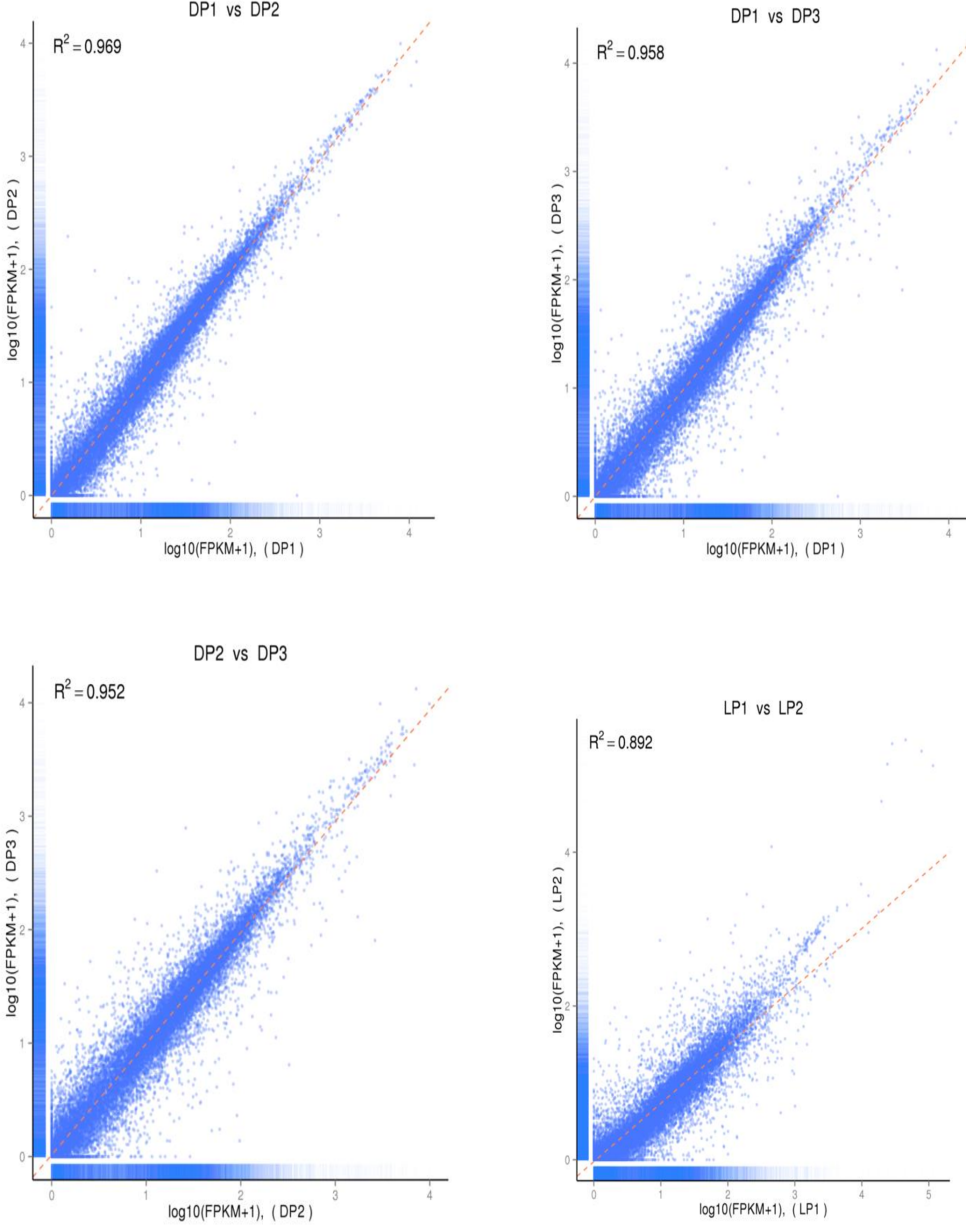
A scatter plot of the correlation analysis.

The correlation of transcript expression between samples is the most important indicator of the reliability of the experimental results and rationality of the sampling. In the present study, the square of the Pearson correlation coefficient (r^2^) was calculated based on the normalized FPKM values to reflect the correlation of transcript expression between samples. The results indicated that r^2^ ranged from 0.892 to 0.969 between the different samples of each group. All of the correlation values reached 0.80, which means that the selection of every sample in the same group was satisfactory and the results were reliable. In addition, the r^2^ values between the samples of the DP group were as high as 0.92 (an ideal r^2^ value for the best sample-selected condition). The transcript expression of different biological replicates at each stage (dry period and lactation period) is shown in the scatter plot (Fig 3).

### Identification of differentially expressed genes between the DP and LP

**Fig 4:**
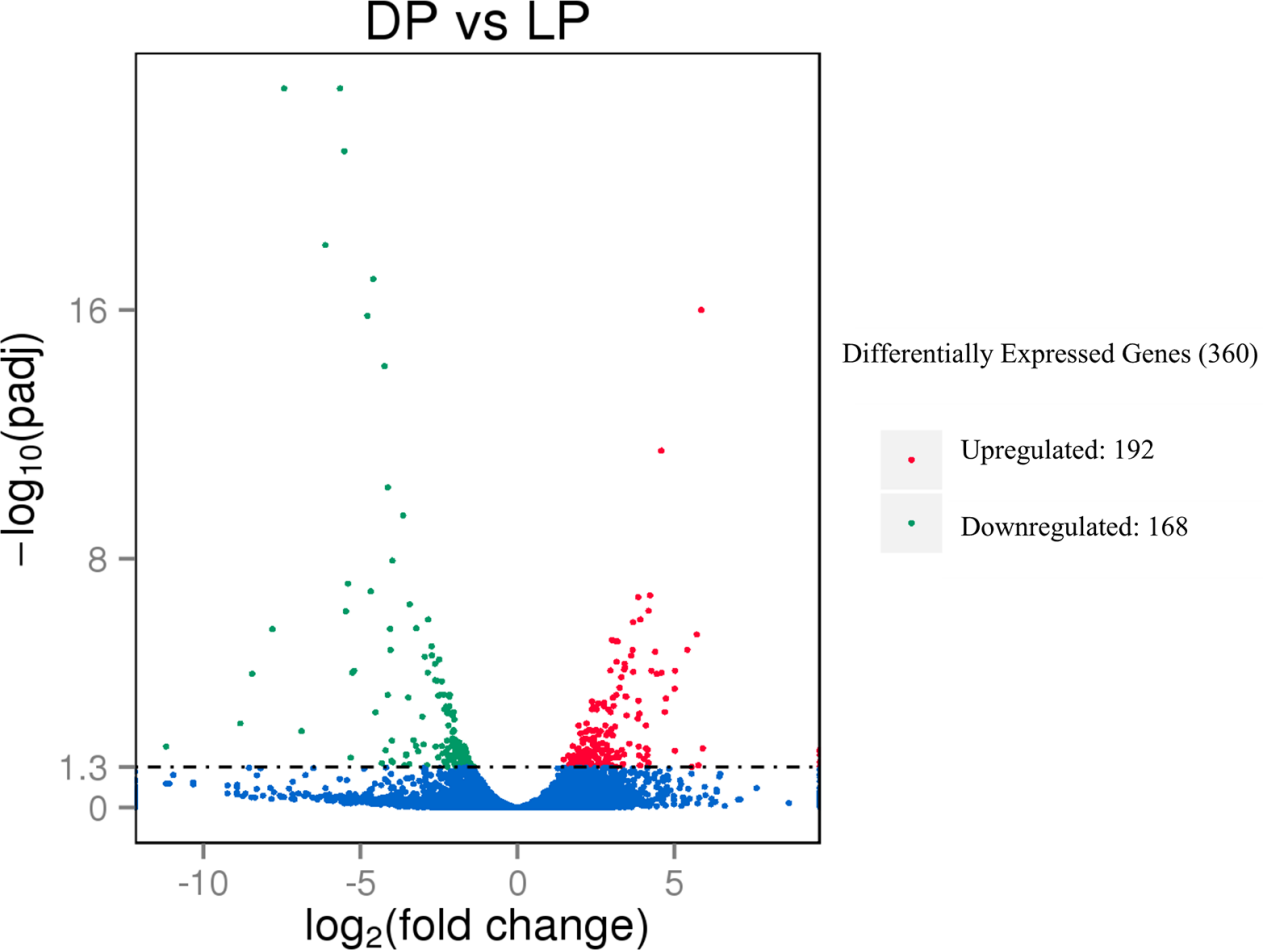
A volcano plot of differentially expressed genes between two groups.

To better survey the regulatory mechanism of lactogenesis, milk secretion and mammary gland development, it is important to identify the differentially expressed genes of mammary gland tissue between the dry period (DP) and lactation period (LP). The DESeq R package (1.18.0) was used to perform a differential expression analysis. The results indicated that 360 differentially expressed genes were detected between the DP and LP when the adjusted P value (padj < 0.05) was used as a cutoff value. Of these, 192 genes were upregulated while 168 genes were downregulated in the yak mammary gland tissue of the dry period compared to that of the lactation period. The distribution of the differentially expressed genes is shown in a volcano plot (Fig 4).

**Fig 5:**
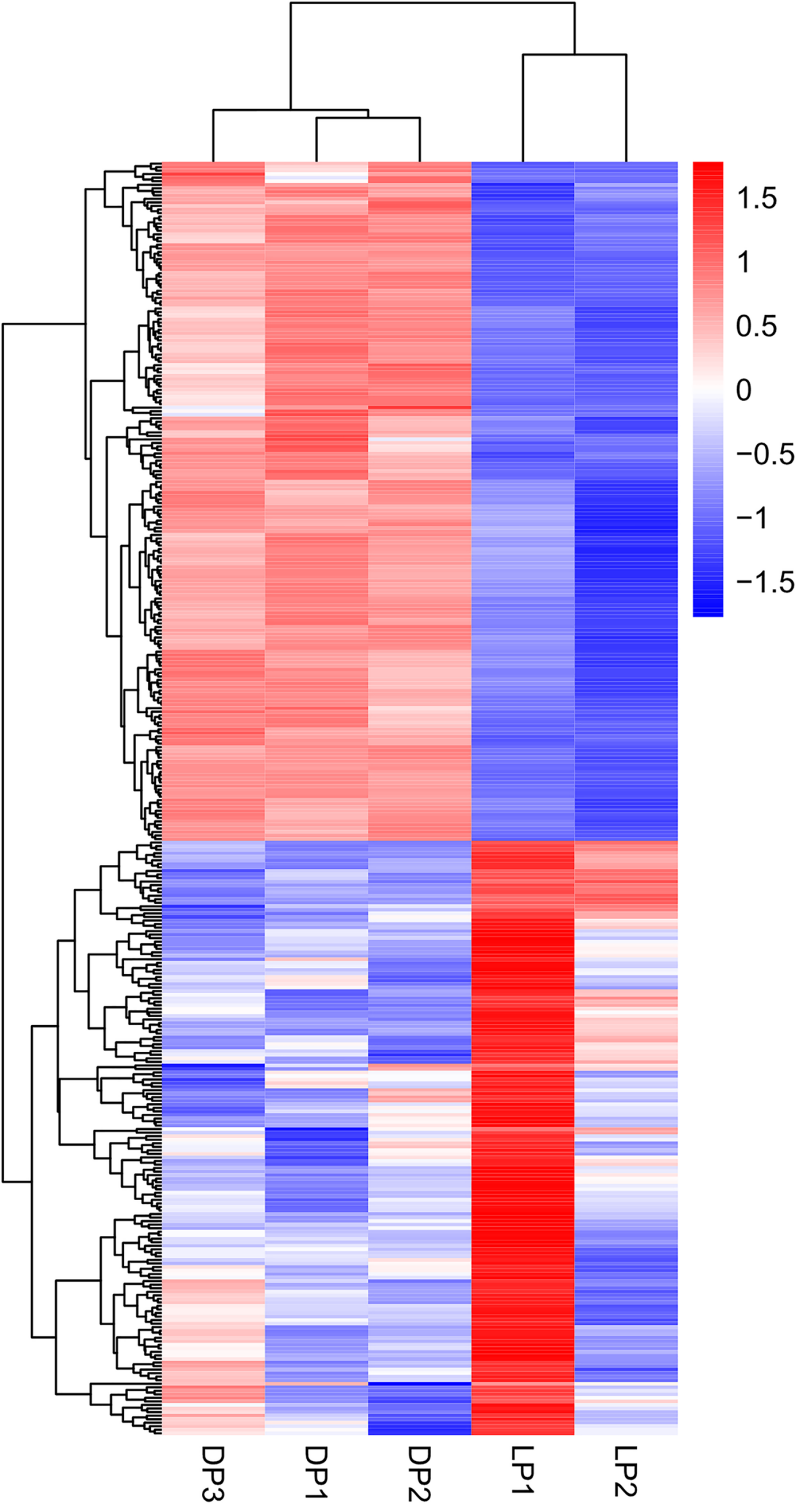
Clustering of the differentially expressed genes in the yak mammary gland during the two periods. Columns indicate individual samples, rows represent each differentially expressed gene, and the color scale represents the relative expression level of the differentially expressed genes.

In addition, a systematic cluster analysis of the differentially expressed genes was performed to gain further insight into the similarities at the transcriptome scale among the yak mammary tissues obtained from the dry period and lactation period. The results indicated that the individual samples belonging to the same group were clustered together; furthermore, the patterns based on the relative expression levels of differentially expressed genes had obvious differences between the two groups (Fig 5). These results implied that the samples used in the present study were selected appropriately and the grouping of these samples was reasonable.

### GO enrichment analysis of differentially expressed genes

**Fig 6:**
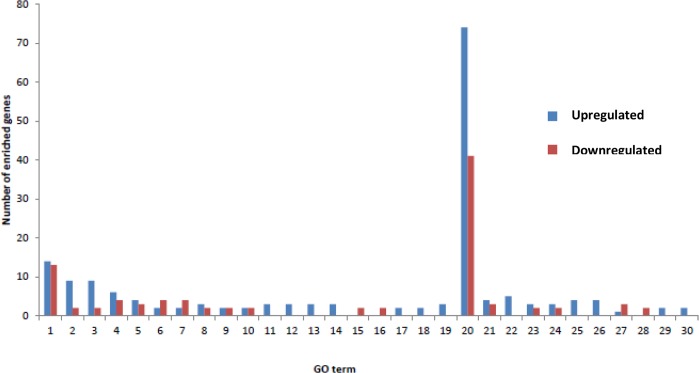
The most enriched 30 GO terms (DP vs LP). Biological Process: 1. Multi-organism process; 2. Immune response; 3. Immune system process; 4. Microtubule-based process; 5. Transition metal ion transport; 6. Regulation of cytoskeleton organization; 7. Regulation of organelle organization; 8. Ferrous iron transport; 9. Regulation of microtubule-based process; 10. Regulation of microtubule cytoskeleton organization; 11. Binding of sperm to the zona pellucida; 12. Cell recognition; 13. Cell-cell recognition; 14. Sperm-egg recognition; 15. Polysaccharide transport; 16. Polysaccharide localization; 17. Plant-type cell wall organization; 18. Plant-type cell wall organization or biogenesis.Cellular Component: 19. Anchored component of membrane. Molecular Function: 20. Protein binding; 21. Transition metal ion transmembrane transporter activity; 22. Cytokine activity; 23. Ferrous iron transmembrane transporter activity; 24. Iron ion transmembrane transporter activity; 25. Chemokine activity; 26. Chemokine receptor binding; 27. Kinase binding; 28. Polysaccharide transmembrane transporter activity; 29. Plastoquinol—plastocyanin reductase activity; 30. Oxidoreductase activity, acting on diphenols and related substances as donors, with copper protein as the acceptor.

A Gene Ontology (GO) enrichment analysis of 360 differentially expressed genes was implemented by the GOseq R package. The differentially expressed genes were categorized into three major functional types: biological process, molecular function, and cellular component. The top GO terms of enriched genes were protein binding, multi-organism process, immune system, and immune response, in which 115, 27, 11, and 11 genes were distributed, respectively. We show the most enriched 30 GO terms in a histogram (Fig 6). The number of upregulated and downregulated genes of every GO term in the DP compared with the LP is also reflected in the histogram.

### KEGG enrichment analysis of differentially expressed genes

**Fig 7:**
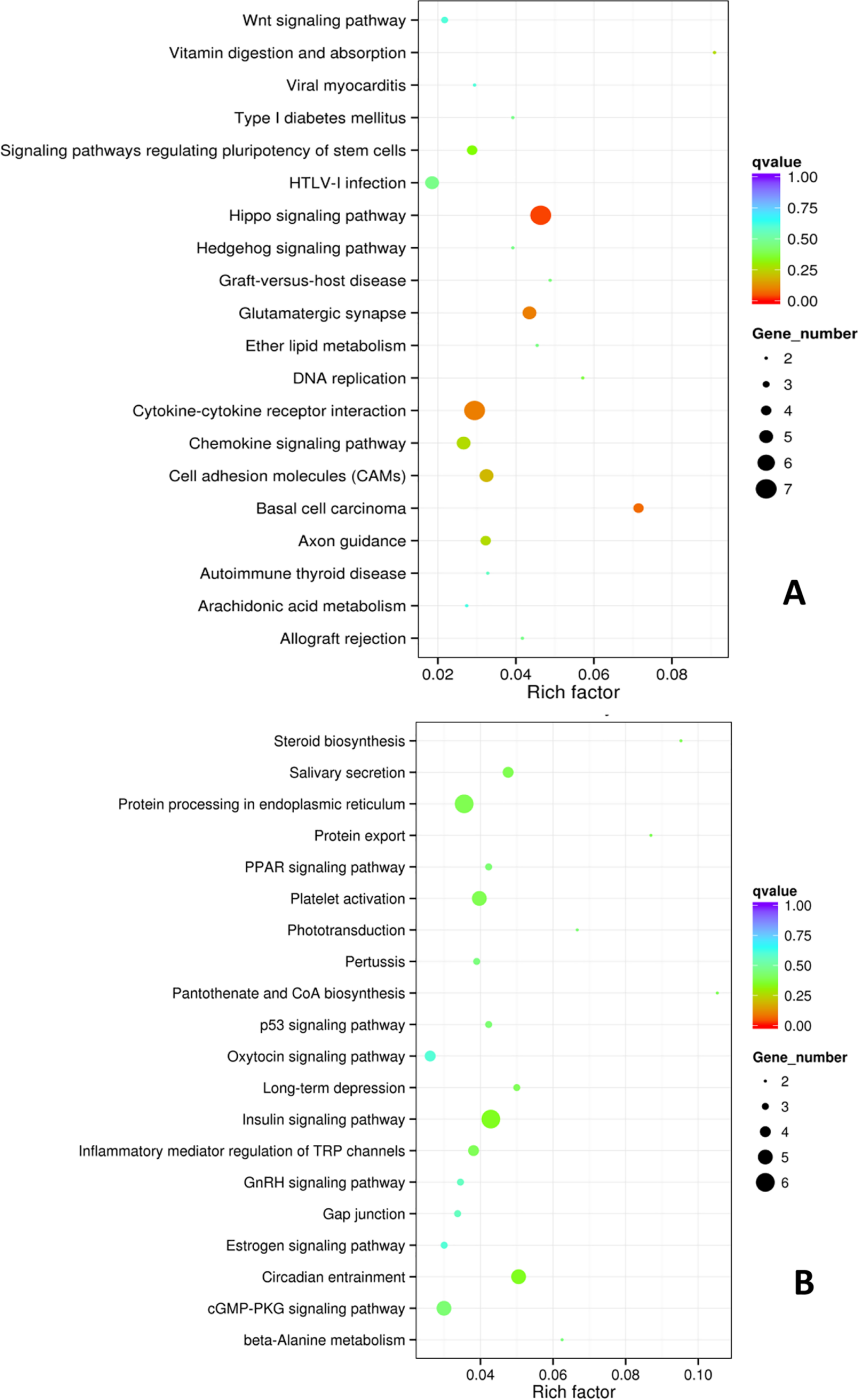
The KEGG Pathway Enrichment of up- (A) and Downregulated Genes (B) in DP Compared with the LP.

KOBAS software was used to test the statistical enrichment of the differential expression of genes in KEGG pathways. In the yak mammary gland tissue of the DP compared with that of the LP, 192 upregulated genes were involved in 106 KEGG pathways; furthermore, 168 downregulated genes were involved in 135 KEGG pathways. The most enriched 20 KEGG pathways of up- and downregulated genes are shown in a scatter diagram (Fig 7). The diagram indicates that the upregulated genes were mostly enriched in the Hippo signaling, basal cell carcinoma, glutamatergic synapse, cytokine-cytokine receptor interaction, cell adhesion molecules (CAMs), vitamin digestion and absorption, axon guidance, chemokine signaling pathway, signaling pathways regulating pluripotency of stem cells, DNA replication, and graft-versus-host disease KEGG pathways; furthermore, the downregulated genes were mostly enriched in the insulin signaling, circadian entrainment, protein processing in the endoplasmic reticulum, platelet activation, salivary secretion, pantothenate and CoA biosynthesis, steroid biosynthesis, protein export, inflammatory mediator regulation of TRP channels, long-term depression, cGMP-PKG signaling pathway, PPAR signaling, p53 signaling and other KEGG pathways. In addition, although not significant, we identified two downregulated genes enriched in the mTOR signaling pathway (P Value = 0.14).

## Discussion

The mammary gland is a dynamic organ that undergoes structural, physiological and functional changes during the female reproductive cycle. It can be divided into different periods according to the physiological processes of the mammary gland. In the dry period, a large-scale degenerating change occurs and lactation activity gradually ceases. The physiological processes within the mammary gland at this period include the following: a degenerating change in the cellular architecture occurs during the first days of active involution, possibly caused by the high intra-alveolar pressure. Then, extensive histological changes affect the synthesis of the milk constituents. During the steady state of involution, there is a loss of integrity of the mammary epithelium, and various substances enter the mammary lumen from the blood [22]. In addition, the dry period is essential for the mammary gland to regenerate after the previous lactation. In addition, it is also necessary to render an appropriate milk yield during the subsequent period [23]. On the other hand, the mammary gland is characterized by extensive mammogenesis and lactogenesis in the lactation period [9]. Therefore, it is obvious that these two periods provide perfect models for the comparative analysis of mammary gland development and lactation. In the present study, five yaks, of which three were in the dry period and two were in the lactation period, were selected as the object of research to investigate differentially expressed genes. The selection and grouping of these animals was reasonable and meaningful. We also constructed two types of cDNA sequence libraries using yak mammary tissues of the dry period (DP) and lactation period (LP) for the first time. In view of the differentially expressed genes between the two periods and the subsequent correlation and clustering analyses, the results of the animal grouping are reliable and accurate.

The most important aspect of mammary gland research must involve investigating lactation-related gene expression. Gene expression profiling of mammary tissue is a powerful tool that has been used to catalog and characterize the role of genes expressed during lactation. In the past few years, the transcriptome has been widely analyzed using microarrays [24–28]. In recent years, however, next-generation sequencing (NGS) technology has been used to explore differentially expressed genes in the mammary gland tissue or milk under different physiological and pathological conditions [5,17,29–31]. This research has revealed that an abundance of genes plays a very complex and important role in the regulation of lactation and mammary gland tissue health. Cui et al. [29] generated a bovine transcriptome from the mammary glands of four lactating Holstein cows with extremely high and low phenotypic values of milk protein and fat percentage using RNA-seq techniques. They obtained 48,967,376~75,572,578 uniquely mapped reads that covered 82.25% of the annotated transcripts. Among them, 31 differentially expressed genes (p < 0.05, false discovery rate q < 0.05) between the high and low groups of cows were revealed. Gene ontology and pathway analyses demonstrated that the 31 differentially expressed genes were enriched in specific biological processes with regard to protein metabolism, fat metabolism, and mammary gland development (p < 0.05). An integrated analysis of differential gene expression indicated that TRIB3, SAA (SAA1, SAA3, and M-SAA3.2), VEGFA, PTHLH, and RPL23A were the most promising candidate genes affecting milk protein and fat percentage. However, there has been no similar report about transcriptome research of the mammary gland in yaks using RNA-seq techniques. The present study has described the comprehensive mammary gland transcriptome of the yak during the dry period (DP) and lactation period (LP) for the first time. cDNA sequence libraries of the dry period (DP) and lactation period (LP) were constructed. A total of 45,423,478 to 53,274,976 clean reads were obtained and 76.85% to 84.56% of the total reads were aligned to the BosGru_v2.0 (https://www.ncbi.nlm.nih.gov/assembly/GCF_000298355.1/) yak reference genome. In addition, 74.72% to 80.65% of the reads were uniquely mapped to the yak genome for each sample. Compared with reports for the non-model organisms yak [32], sheep [33], pigs [34] and bovine [35], in which 61.4%~75.0% of reads mapped to the reference genome, our mapping percentage was almost comparable or better, which ensures a reliable downstream analysis. This study provides valuable materials for elucidating the molecular mechanisms of lactation regulation in the yak.

During the different stages of lactation, there are distinct changes of mammary tissue development and function. The dry period (DP) and lactation period (LP) are two entirely different stages of mammary gland development during the lactation cycle. To better survey the molecular mechanism of lactation, it is important to identify the differentially expressed genes (DE genes) between the two stages. In the yak mammary gland, 360 DE genes were detected between the two groups when the adjusted P value (padj < 0.05) was used as a cutoff value. Our gene ontology analyses indicated that the DE genes were annotated in different functional types. In the yak mammary gland tissue of the dry period compared to the lactation period, some downregulated genes were most enriched in the biological process that was related to the biosynthesis of milk components, such as the GO terms of polysaccharide transport (two genes were downregulated while 0 were upregulated) and polysaccharide localization (two genes were downregulated while 0 were upregulated). In addition, some downregulated genes were enriched in the biological process that was related to cell proliferation, such as the GO terms of regulation of cytoskeleton organization (four genes were downregulated while two were upregulated) and regulation of organelle organization (four genes were downregulated while two were upregulated). These results suggest that there is an adaptation in the mammary gland at the transcriptional level in the dry period and many genes that are involved in lactogenesis, lactation and mammary gland development are inactivated.

Furthermore, KEGG pathway analysis of the DE genes provided significant insight into the potential biological pathways activated or inhibited in response to lactogenesis and lactation in the yak mammary gland. In the present study, the strongly impacted KEGG pathways were ‘Hippo signaling pathway’, ‘Cytokine-cytokine receptor interaction’, ‘Cell adhesion molecules (CAMs)’, ‘Vitamin digestion and absorption’, ‘Insulin signaling pathway’, ‘Protein processing in endoplasmic reticulum’, ‘Steroid biosynthesis’, ‘Protein export’, ‘PPAR signaling pathway’, ‘p53 signaling pathway’, ‘GnRH signaling pathway’, ‘Oxytocin signaling pathway’, and ‘Estrogen signaling pathway’. These data may provide candidate genes with a high probability of having functional roles in regulating the change between the dry period and lactation period in the yak mammary gland.

Although the Hippo pathway has complex characteristics and a cross-linking dialogue with other signaling pathways, it has been acknowledged to play an important role in the regulation of organ size, cell cycle, apoptosis and maintenance of homeostasis. In response to high cell densities, activated LATS1/2 phosphorylates the transcriptional coactivators YAP and TAZ, promoting their cytoplasmic localization, leading to cell apoptosis and restricting organ size overgrowth. When the Hippo pathway is inactivated at low cell density, YAP/TAZ translocates into the nucleus to bind to the transcription enhancer factor (TEAD/TEF) family of transcriptional factors to promote cell growth and proliferation [36]. Recent findings suggested that the Hippo pathway regulates female reproductive system development [37–39] and tumorigenesis [40,41]. Therefore, more and more researchers pay attention to Hippo pathway research. Aside from research related to breast cancer [42–44], the physiological role of Hippo signaling in mammary gland development remains poorly understood. Chen et al. [45] used mouse genetics to interrogate Hippo signaling in the mammary gland in vivo. The results indicated that the Hippo pathway is genetically dispensable in virgin mammary glands but specifically required during pregnancy when mammary epithelia undergo rapid growth and terminal differentiation. In the present study, the results of KEGG pathway analysis indicate that the most enriched pathway term is Hippo signaling. A total of seven DE genes that belong to this pathway were upregulated in the yak mammary gland of the dry period compared to the lactation period. This implies that the development of the yak mammary gland is probably inhibited through the Hippo pathway during the dry period in the yak.

As an important pathway for DE genes, the insulin signaling pathway was enriched by downregulated genes in the yak mammary gland tissue of the dry period compared with the lactation period. Insulin is known to be an important regulator of milk secretion in the lactating mammary gland. Neville et al. [46] examined the role of insulin signaling in mammary development in the mouse. They found that the insulin receptor (IR) plays an important role both in alveolar development and differentiation of the mouse mammary gland. Their experiments conducted on mammary epithelial cells (MECs) isolated from pregnant mice have also verified that insulin can stimulate lumen formation, mammary cell size, acinar size, acinar casein content, and the formation of lipid droplets. Using an array analysis, they found significant downregulation of differentiation-specific genes and upregulation of cell cycle and extracellular matrix genes. Therefore, they believe that insulin stimulates differentiation and may inhibit cell proliferation in the mammary gland of the mid-pregnant mouse. Recently, Cohick [47] summarized the available data that address a role for insulin in the secretory differentiation of the mammary gland. Abundant data expanded our understanding of the role for insulin in functional differentiation that includes both transcriptional and post-transcriptional regulation of multiple genes involved in the development of the mammary gland and lactation process. In the present study, insulin signaling is also the most enriched pathway term of the downregulated DE genes in the dry period yak mammary gland compared with the lactation period. There are six genes involved in this pathway. These genes are related to fatty acid biosynthesis, gluconeogenesis and protein synthesis and proliferation/differentiation processes, such as FBP1 (fructose-bisphosphatase 1, Novel02167, log2 fold change = -5.3093), CALM (calmodulin, ENSP00000403491-D4, log2 fold change = -1.6542, ENSP00000379210-D7, log2 fold change = -1.7085), RPS6 (ribosomal protein S6, ENSBTAP00000045373-D8, log2 fold change = -1.7901), EIF4EBP1 (eukaryotic translation initiation factor 4E binding protein 1, ENSBTAP00000039615-D1, log2 fold change = -2.1687), and PTPRF (protein tyrosine phosphatase receptor type F, ENSBTAP00000024046-D1, log2 fold change = -1.6001). These data may provide candidate genes with a high probability of having functional roles in regulating lactogenesis and mammary gland development in the yak.

In the present study, steroid biosynthesis and the mTOR signaling pathway were also found to be enriched by downregulated genes in the yak mammary gland of the dry period compared with the lactation period. The important role of steroids for mammary gland growth and development has been commonly accepted by many researchers [48]. Recently, Shu et al. [8] found that the pathway of steroid biosynthesis was upregulated from late pregnancy to peak lactation in the porcine mammary gland.

In addition, a master role for mTOR in the regulation of protein synthesis, particularly translation, in all tissues of mammals has been well defined [49]. Several lines of evidence support a pivotal role for mTOR in milk protein synthesis in mice and cows [50–53]. As described previously, the networks of factors involved in the regulation of milk protein synthesis in the bovine include the mTOR signaling pathway [53]. Similar data that highlighted a role for mTOR in the regulation of milk protein synthesis were also obtained both in rodents [54] and ruminants [55]. Recently, a phenomenon of mTOR pathway activation was reported in the transition of the porcine mammary gland [8]. Our results are consistent with this previous research. Therefore, we believe that the inhibition of the synthesis of milk constituents such as fatty acids and milk protein is probably related to the downregulation of steroid biosynthesis and the mTOR signaling pathway in the yak mammary gland during the dry period compared with the lactation period.

In summary, lactogenesis, milk secretion and mammary gland development are very complex processes. A large number of genes are involved in the regulation of these processes. Investigations of the differential expression of genes between different physiological stages of the mammary gland only provide a way to identify candidate genes with a high probability of having functional roles in these processes. Further analyses of these gene functions are necessary for us to have a complete understanding of the molecular mechanism of lactation regulation.

## Conclusions

This study investigated the character of the mammary gland transcriptome in the yak using RNA-seq. Two types of cDNA sequence libraries were constructed using the yak mammary tissues of a dry period (DP) and a lactation period (LP). Then, a total of 360 differentially expressed genes were detected between the two groups when the adjusted P value (padj < 0.05) was used as a cutoff value. Although the functions of many of these DE genes are not yet thoroughly elucidated in the yak mammary gland, our GO and KEGG analysis of the up- and downregulated genes provides important insights into the molecular events involved in lactogenesis, lactation and mammary gland development and will guide further research to enhance milk yield and optimize the constituents of yak milk.
